# A CENP-S/X complex assembles at the centromere in S and G2 phases of the human cell cycle

**DOI:** 10.1098/rsob.130229

**Published:** 2014-02-12

**Authors:** Carsten Dornblut, Nadine Quinn, Shamci Monajambashi, Lisa Prendergast, Chelly van Vuuren, Sandra Münch, Wen Deng, Heinrich Leonhardt, M. Cristina Cardoso, Christian Hoischen, Stephan Diekmann, Kevin F. Sullivan

**Affiliations:** 1 Molecular Biology, FLI, Beutenbergstrasse 11, Jena 07745, Germany; 2 Centre for Chromosome Biology, National University of Ireland, Galway, Galway, Ireland; 3 Department of Biology II, Center for Integrated Protein Science, Ludwig Maximilians University Munich, Planegg-Martinsried, Munich 82152, Germany; 4 Department of Biology, Technische Universität Darmstadt, Darmstadt 64287, Germany

**Keywords:** centromere, mitosis, constitutive centromere-associated network, kinetochore

## Abstract

The functional identity of centromeres arises from a set of specific nucleoprotein particle subunits of the centromeric chromatin fibre. These include CENP-A and histone H3 nucleosomes and a novel nucleosome-like complex of CENPs -T, -W, -S and -X. Fluorescence cross-correlation spectroscopy and Förster resonance energy transfer (FRET) revealed that human CENP-S and -X exist principally in complex in soluble form and retain proximity when assembled at centromeres. Conditional labelling experiments show that they both assemble *de novo* during S phase and G2, increasing approximately three- to fourfold in abundance at centromeres. Fluorescence recovery after photobleaching (FRAP) measurements documented steady-state exchange between soluble and assembled pools, with CENP-X exchanging approximately 10 times faster than CENP-S (*t*_1/2_ ∼ 10 min versus 120 min). CENP-S binding to sites of DNA damage was quite distinct, with a FRAP half-time of approximately 160 s. Fluorescent two-hybrid analysis identified CENP-T as a uniquely strong CENP-S binding protein and this association was confirmed by FRET, revealing a centromere-bound complex containing CENP-S, CENP-X and CENP-T in proximity to histone H3 but not CENP-A. We propose that deposition of the CENP-T/W/S/X particle reveals a kinetochore-specific chromatin assembly pathway that functions to switch centromeric chromatin to a mitosis-competent state after DNA replication. Centromeres shuttle between CENP-A-rich, replication-competent and H3-CENP-T/W/S/X-rich mitosis-competent compositions in the cell cycle.

## 2. Introduction

The centromere/kinetochore complex guides chromosome movements and cell-cycle progression through spindle microtubule interactions in dividing cells [1,2]. These functions are determined by a differentiated chromatin domain that contains CENP-A, a conserved centromere-specific variant of histone H3 [3–5]. CENP-A itself plays a role in propagating centromere identity as well as nucleating kinetochore formation [6–8]. Both of these functions are supported by a group of tightly associated chromatin proteins constitutively present at centromeres, collectively known as the constitutive centromere-associated network (CCAN) [9–12]. CCAN subunits contribute to the assembly of CENP-A chromatin and establishment of the kinetochore [12–17].

A distinctive chromatin assembly process maintains centromeric chromatin. In vertebrates and fungi, a conserved CENP-A chaperone, HJURP or Scm3, functions in CENP-A deposition [18–21]. CENP-A assembly in human cells is uncoupled from DNA replication and takes place principally in G1, initiated by post-mitotic association of the Mis18 complex and HJURP with centromeres [7,18,19,22–24]. G1 phase events include CENP-A deposition [25], exchange of histone H3.3 nucleosomes incorporated in the prior cell cycle [26] and the action of the RSF complex, which converts loosely bound CENP-A into a form with nucleosome-like solubility properties in mid-G1 [27]. At the end of G1, CENP-A is fully restored and an MgcRacGAP-dependent reaction stabilizes the newly incorporated CENP-A nucleosomes prior to S phase [28]. During the ensuing S phase, CENP-A is distributed to daughter chromatids, where it exhibits stable association over multiple generations [24,25,29].

During S phase, histone H3-containing nucleosomes are deposited at the centromere, presumably occupying nucleosomal sites vacated by CENP-A [26]. In addition, a distinct heterotetrameric nucleoprotein particle, formed by CENPs -T, -W, -S and -X, is found at centromeres where it functions as a chromatin-based receptor for the Ndc80 complex, the principal microtubule-binding protein of the kinetochore [30–33]. CENP-T and -W assemble in late S and G2 phases of the cell cycle, a process that is required for proper kinetochore assembly in each cell cycle [14]. The CENP-S/-X complex is itself not essential for mitosis in DT40 cells but plays a role in stabilizing kinetochore structure [34]. In addition to their centromeric function, CENP-S and -X function in association with FANCM in the DNA damage response [35–37]. To understand their role in kinetochore assembly, we used biophysical and cell biological methods to investigate the modes of binding of CENP-S and -X to both types of sites. We show that CENP-S and -X exist in complexed form in the nucleoplasm and at kinetochores and that their binding to the centromere is mechanistically distinct from binding to DNA damage sites. The CENP-S/-X complex assembles in S and G2 phases by an open exchange mechanism into a structure that includes CENP-T in close proximity to histone H3. These results confirm the existence of the novel CENP-T/W/S/X particle *in vivo* and show that it assembles at a discrete time in the cell cycle, presumably in the context of post-replicative chromatin. We suggest that, in vertebrates, centromeric chromatin alternates between two functionally distinct states during the cell cycle, using different configurations of chromatin subunits at a single locus to promote centromere (in G1) and kinetochore function (in G2), respectively.

## 3. Results

### 3.1. CENPs -S and -X are complexed together in soluble and assembled states in living cells

**Figure 1.**
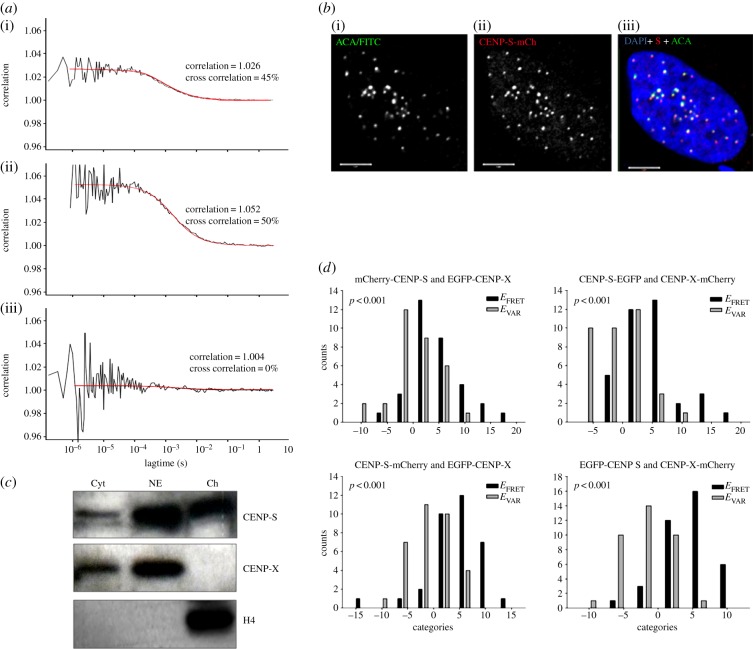
CENP-S and CENP-X exist primarily in a heterotypic complex in solution and at centromeres. (*a*) FCCS analysis of EGFP-mRFP fusion protein (i), EGFP-CENP-X and mCherry-CENP-S (ii) and EGFP + mRFP (iii) in the cell nucleus. The cross-correlation function (black line) results from the correlation of the autocorrelation functions of EGFP and mCherry/mRFP as described in the text, while the red line shows the fitted curve derived from the cross-correlation values. The number of co-localized molecules for EGFP-CENP-X + mCherry-CENP-S (50%) and the positive control (45%) is similar, indicating CENP-S/-X co-localization. (*b*) Immunofluorescence confirms that CENP-FP constructs target normally to centromeres. U2OS cells transfected with CENP-S-mCherry were immunostained with anticentromere antibodies (i), revealing co-localization with CENP-S-mCherry (ii) within the nucleus (DAPI; (iii)). Comparable results were observed with CENP-X-FP constructs. (*c*) Partitioning of CENP-S and CENP-X in the nucleus and cytoplasm. Cytoplasmic proteins (Cyt) were separated from nuclei, which were further extracted with 0.35 M NaCl to generate a nuclear extract (NE) and chromatin-bound (Ch) fractions and probed by western blot to detect CENP-S, CENP-X and histone H4. (*d*) FRET analysis reveals the association of the CENP-S/CENP-X complex at centromeres.

CENP-S and -X have been reported to co-purify and to form tetrameric complexes with themselves as well as with CENPs -T and -W [30,34], suggesting stable complex formation. Here, the presence of such a complex *in vivo* was probed by fluorescence cross-correlation spectroscopy (FCCS), using EGFP-CENP-X and mCherry-CENP-S co-expressed in U2OS cells (figure 1*a*,*b*; electronic supplementary material, figure S1). Tagged proteins were introduced by transient transfection and exhibited centromeric targeting independently of the site of FP fusion and were used to model CENP-S and -X behaviour in cells (see electronic supplementary material, figure S1). Experiments with control fluorochromes in monomeric (EGFP + mRFP) or fused (mRFP–EGFP) forms established the dynamic range of this system (figure 1*a*). Co-diffusion is detected as described by Bacia & Schwille [38]. EGFP + mRFP measurements resulted in a cross-correlation coefficient (cc) of 1.004, indicating 0% co-diffusing molecules, whereas mRFP-EGFP resulted in cc = 1.027, indicating 45% co-diffusion. This low value is most likely due to partial maturation of mRFP, which results in a heterogeneous population containing mono- and difluorescent molecules [39]. For the CENPs, measurements of EGFP-CENP-X and mCherry-CENP-S in the nucleoplasm show individual autocorrelation values of 1.126 (EGFP) and 1.104 (mCherry). Cross-correlation measurements resulted in a cc = 1.052, indicating that 50% of the molecules are co-migrating (figure 1*a*). In total, 19 FCCS measurements were carried out in the nucleoplasm, all indicating that at least 30–50% of EGFP-CENP-X and mCherry-CENP-S are co-diffusing and thus co-resident in a single complex. Similar values were obtained in 12 cytoplasmic FCCS measurements. By contrast, the FCCS analysis of CENP-S association with CENP-T revealed no detectable soluble complex containing these proteins (electronic supplementary material, figure S2). In parallel experiments, biochemical fractionation of HeLa cells revealed that small fractions of both CENP-S and CENP-X are detectable in the cytosol (figure 1*c*). CENP-X was quantitatively extracted from nuclei with 0.35 M NaCl, while histone H4 was quantitatively retained in the chromatin-bound fraction as expected. CENP-S showed intermediate behaviour with more than 50% extracted by 0.35 M NaCl. Taken together, these results show that CENP-S and CENP-X form a soluble co-complex detectable in both nucleus and cytoplasm. The value of 30–50% CENP-S/-X co-diffusion is a minimal estimate owing to RFP maturation behaviour. We conclude that CENP-S and -X exist in a preformed complex similar to other histone fold dimers and that the soluble complex is independent of CENP-T.

*In situ* Förster resonance energy transfer (FRET) analysis was carried out to determine whether the CENP-S/-X complex assembles intact at centromere/kinetochore loci (figure 1*d*). Using the acceptor photobleaching method, we detected FRET between CENP-S-mCherry and EGFP-CENP-X at centromeres. We measured mean *E*_FRET_ values of 4.5 (34 kinetochores in 12 cells) with the control *E*_VAR_ = −0.9 (33 kinetochores in 12 cells), resulting in a difference of 5.4 with *p* < 0.001 (Mann–Whitney rank-sum test). Figure 1*d* shows bar diagrams of FRET measurements between fluorophore-tagged CENP-S and -X. The measured FRET values are listed in table 1. In negative control experiments, unfused EGFP and mCherry, co-transfected in living human cells at similar expression levels, showed no FRET, allowing us to exclude that FRET detected for the protein fusions might be caused by an incidental association of the fluorescent proteins [40]. As a positive control, an EGFP-mCherry hybrid protein was analysed in which both fluorescent proteins are closely connected by a short linker. The mean fluorescence lifetime of EGFP within eight nuclei was significantly decreased, indicating that FRET occurred between the two fluorophores with a FRET efficiency of 15%. This value is in good quantitative agreement with the results of Tramier *et al*. [41]. We observed FRET between CENP-S and CENP-X irrespective of termini at which these proteins were tagged, confirming *in vitro* data [30,34], being consistent with a stable heterodimeric or higher order complex at centromeres.

**Table 1.** Summary of FRET measurements in this study. Summary of FRET measurements between CENP-S and CENP-A, CENP-R, CENP-T, CENP-X and nucleosome H3.1, respectively. CENP-S-mCherry and EGFP-CENP-T were measured twice. *N*_kin_ is the number of analysed kinetochores in a bleached area. *E*_FRET_ is the mean value of difference in donor-fluorescence after acceptor-bleach in a bleached region of the nucleus. *E*_VAR_ is the mean value of difference in donor-fluorescence after acceptor-bleach in an unbleached region of the nucleus. Δ*E* = *E*_FRET_ – *E*_VAR_. *p*-value obtained from Mann–Whitney rank-sum test. ++, positive FRET; +, non-significant FRET; −, no FRET.

proteins	measured variant	analysed termini	*N*_kin_	*E*_FRET_	*E*_VAR_	Δ*E*	*p*-value	FRET?
CENP-S CENP-R	EGFP-S mCh-R	N–N	35	−0.857	−0.703	−0.154	0.962	−
	S-mCh R-EGFP	C–C	37	1.027	−0.700	1.727	0.093	−
	S-mCh EGFP-R	C–N	38	0.947	−0.105	1.053	0.471	−
	mCh-S R-EGFP	N–C	31	0.065	−1.125	1.190	0.394	−
CENP-S CENP-T	EGFP-S mCh-T	N–N	40	1.900	−0.190	2.090	0.067	−
	S-EGFP T-mCh	C–C	39	4.154	−0.341	4.495	<0.001	++
	S-mCh EGFP-T	C–N	47	2.851	0.118	2.733	0.027	+
	S-mCh EGFP-T	C–N	29	0.345	−1.412	1.757	0.142	−
	EGFP-S T-mCh	N–C	32	3.000	−1.250	4.495	<0.001	++
CENP-S CENP-X	mCh-S EGFP-X	N–N	33	4.788	0.250	4.538	<0.001	++
	S-EGFP X-mCh	C–C	36	4.778	−0.778	5.556	<0.001	++
	S-mCh EGFP-X	C–N	34	4.471	−0.909	5.380	<0.001	++
	EGFP-S X-mCh	N–C	38	4.421	−2.000	6.421	<0.001	++
CENP-S H3.1	EGFP-S H3.1-mCh	N–C	50	2.880	−1.360	4.240	<0.001	++
	S-EGFP H3.1-mCh	C–C	47	2.085	−1.565	3.650	<0.001	++
CENP-S CENP-A	S-EGFP A-mCh	C–C	37	1.351	−1.368	2.720	0.008	+
	EGFP-S A-mCh	N–C	38	0.737	−0.100	0.837	0.268	−

### 3.2. Relative abundance of CENP-S/-X through the cell cycle

**Figure 2.**
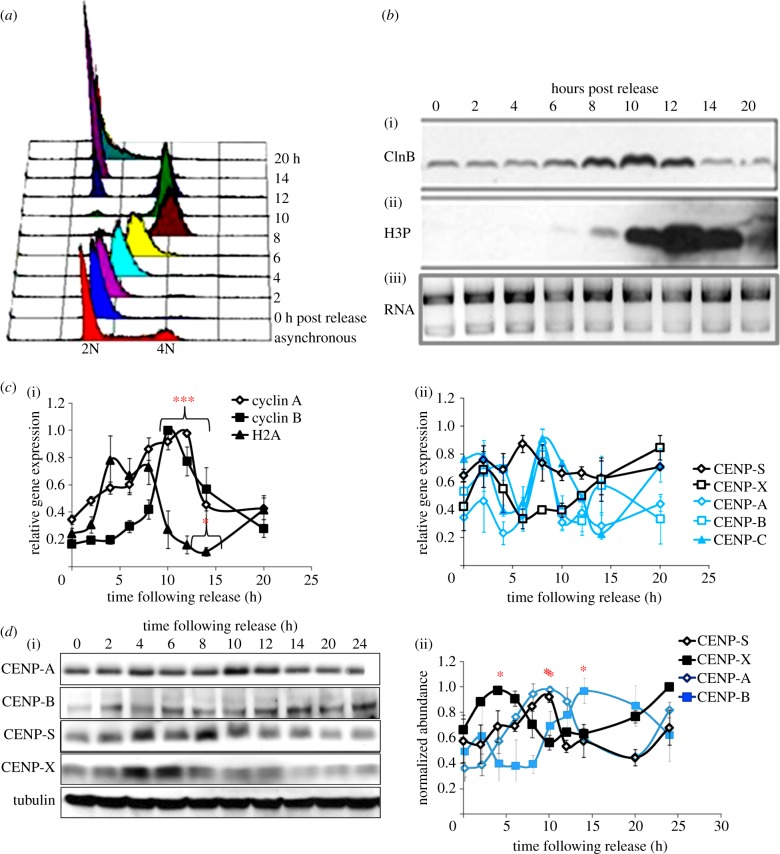
Cell-cycle regulation of CENP-S and CENP-X gene products in HeLa cells. HeLa cells were fractionated across the cell cycle using a double-thymidine protocol. Samples were taken at intervals for quality control and analysis of CENP regulation. (*a*) Flow cytometry confirms synchronous progression of cells through the cell cycle following release from thymidine block. (*b*) Western blot analysis with cyclin B (i) and Ser10-phospho histone H3 (ii) demonstrates normal progression through mitosis and into G1 following release. RNA (iii) isolated from each cell-cycle time point was analysed by agarose gel electrophoresis. (*c*) Quantitative reverse-transcript PCR was used to examine the relative abundance of control genes cyclin A, cyclin B and histone H2A (i), documenting normal cell-cycle regulation of these transcripts. CENP-S and CENP-X transcripts (ii) showed no significant modulation during the cell cycle. (*d*) Western blot analysis of cell cycle fractions (i) shows that CENP-S and CENP-X are uncoordinated in abundance across the cell cycle, with CENP-S maximal in G2 in parallel with CENP-A (ii). Statistical significance of signal at each time point was measured by one way analysis of variance (ANOVA) followed by a Dunnett's multiple comparison versus the 4 h time point; *p ≤ 0.01, ***p ≤ 0.001.

The presence of a CENP-S/-X complex in the cytoplasm and nucleoplasm suggests that both proteins assemble at centromeres as a unit consisting of at least a histone fold dimer. In order to examine the assembly properties of this complex during the cell cycle, we first examined the relative abundance of transcripts encoding the two subunits as well as other CCAN components using qRT-PCR. HeLa cell populations were synchronized with the double-thymidine protocol and sampled following release into the cell cycle (figure 2*a*). Western blot analysis of fractions with cyclin B and histone H3-phospho-Ser10 antibodies confirmed cell-cycle progression through mitosis and into G1 (figure 2*b*). At the RNA level, cyclin A, cyclin B and histone H2A exhibited profiles of transcript abundance across the cell cycle consistent with previous studies [42–44] (figure 2*c*). CENP-A, CENP-B and CENP-C all showed similar trends in their relative transcript abundance with maximum levels reached 8 h after release, when cells are predominantly in G2 phase. By contrast, CENP-S did not exhibit statistically significant modulation in transcript abundance through the cell cycle (figure 2*c*), similar to the other histone fold-containing CCAN members, CENP-T and CENP-W [14]. CENP-X exhibited a trend towards increased expression early in S phase, though this was not statistically significant. While cyclins A and B as well as CENP-C exhibited robust statistically significant modulation across the cell cycle, other transcripts analysed, including CENP-A, did not, suggesting substantial population variation in expression of these transcripts.

The abundance of CENP-S and -X proteins in the HeLa cell cycle was then examined by quantitative western blotting (figure 2*d*). CENP-S reached maximum protein levels 8 h following release, with an approximate 1.5-fold increase across the cell cycle. CENP-X protein increased in a similar fashion but accumulated earlier in S phase with maximum levels reached 4 h after release. In general, there appears to be a trend of downregulation of CCAN protein abundance at mitosis, 12 h following the release from the double-thymidine block, perhaps coupled to proteosome activity, as cells exit mitosis.

### 3.3. Assembly of CENP-S and -X at centromeres during the cell cycle

**Figure 3.**
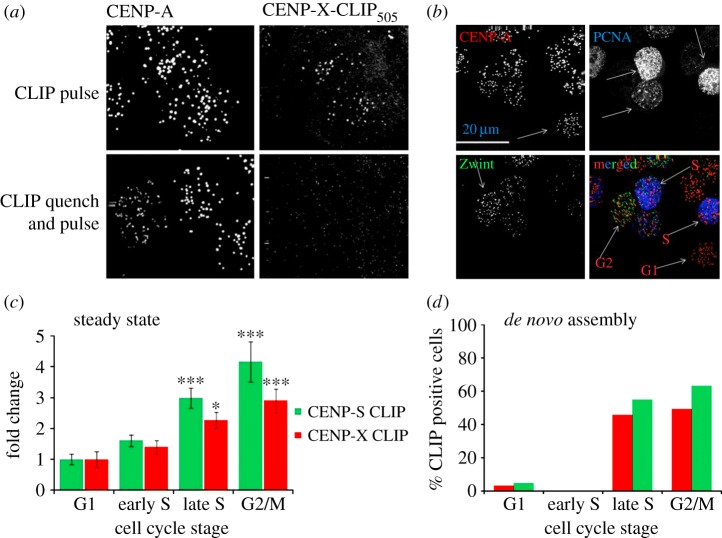
Analysis of CENP-S and CENP-X assembly during the cell cycle. (*a*) CENP-X-CLIP-expressing cells were labelled with CLIP-505 and co-detected with CENP-A by immunofluorescence (top). Pre-treatment of cells with CLIP-BLOCK prevented subsequent labelling with CLIP-505 (bottom). (*b*) Cells were stained with antibodies against PCNA (blue) and Zwint (green) to allow identification of centromeres (CENP-A, red) in defined cell-cycle stages. (*c*) Cells were labelled with CLIP-505 to report steady-state levels of protein and cells were simultaneously stained for cell-cycle classification. Intensity of CLIP-505 is presented as the mean and the standard error of the mean in each of four cell-cycle stages: G1, early S, late S and G2/M. One way analysis of variance (ANOVA) followed by a Dunnett's multiple comparison test was used to analyse the statistical significance of increase in signal intensity relative to G1; *p ≤ 0.01, ***p ≤ 0.001. (*d*) CENP-CLIP fusion proteins were blocked and allowed to recover for 8 h prior to labelling with CLIP-505. They were simultaneously labelled for cell-cycle analysis and the percentage of cells in the population positive for CLIP-505 staining is shown versus cell-cycle stage.

A combined approach was used to investigate the dynamics of assembly of the CENP-S/-X complex at centromeres during the cell cycle. We first used a conditional chemical labelling method (SNAP/CLIP) to examine the abundance and dynamics of tagged derivatives of CENP-S/-X at centromeres in HeLa cells [14,24,45]. HeLa cell lines stably expressing CLIP-tagged derivatives of each protein were established which showed appropriate centromeric targeting of the tagged proteins (figure 3*a*; electronic supplementary material, figure S3) and normal growth kinetics. The conditional CLIP-labelling method allowed us to assay at centromeres: (i) relative abundance in the cell cycle (steady-state labelling) and (ii) the timing of protein assembly (quench-chase-pulse labelling) using microscopic analysis.

The relative steady-state abundance of each subunit at centromeres across the cell cycle was first estimated by direct CLIP-505 labelling in unsynchronized cell populations. Immunofluorescence was used to label centromeres (CENP-A) while cell-cycle phases were established using PCNA and Zwint (figure 3*b*). The intensity of CENP-S-CLIP or CENP-X-CLIP was measured within centromeres, defined by CENP-A staining, and cells were also classified as G1 (no PCNA or Zwint), early or late S phase (PCNA pattern) or G2 or M phases (Zwint). Intensity of CLIP-505 in centromeres was determined from a minimum of 300 centromeres in each of three independently replicated experiments. Average signal intensity values from each stage of the cell cycle were normalized to G1 values to determine the relative abundance of both proteins during the cell cycle. The lowest signal intensities for both CENP-S and CENP-X were found during G1 and early S phase. Both CENP-S and CENP-X showed a significant increase in intensity of three- to fourfold in late S and G2 cells as compared with G1 (figure 3*c*).

A quench-pulse-chase experiment was performed to examine the relative incorporation of newly synthesized CENP-S and CENP-X at centromeres during the cell cycle (figure 3*d*). CLIP-tagged proteins were first blocked in asynchronous populations that were then incubated for a further 8 h to allow incorporation of newly synthesized protein. Cells were then labelled with CLIP-505 and examined by immunofluorescence with CENP-A and cell-cycle markers, as described above. Cells were scored by inspection as either positive or negative for CLIP-505 signal at centromeres. For both subunits, robust CLIP signals were detected in late S and G2 cells, with approximately 50% of these cells being CLIP positive. Less than 5% of G1 cells analysed were positive for CENP-S/-X assembly, while none of the early S-phase cells analysed were positive for either protein (figure 3*d*). Although the labelling window is broad, the lack of labelling in cells in early S phase suggests that these cells do not assemble detectable amounts of CENP-S and -X by this assay. These results are consistent with assembly principally during mid–late S phase which is complete by G2, compatible with the steady-state measurements reported above.

### 3.4. Long-term fluorescence recovery after photobleaching indicates low mobility of CENP-S-EGFP at centromeres in G1, S and G2 phase

**Figure 4.**
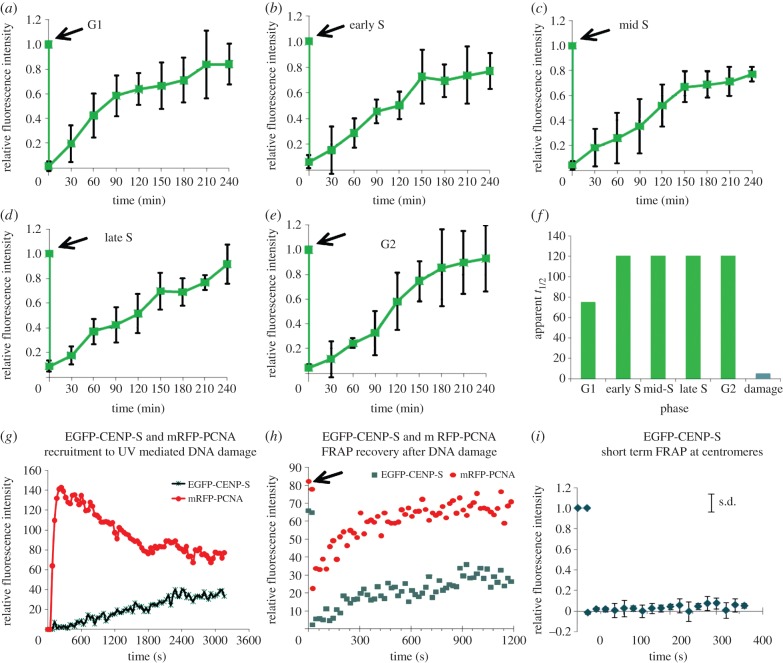
FRAP experiments reveal a dynamic assembly process for CENP-S/X that differs between centromeres and DNA damage sites. CENP-S was transfected into PCNA-mCherry-expressing HeLa cells and subjected to FRAP analysis, plotted as the mean plus and minus the standard deviation of pooled measurements. (*a*–*e*) Long-term FRAP with CENP-S-EGFP at different cell-cycle stages. (*a*) G1; (*b*) early S phase; (*c*) mid-S phase; (*d*) late S phase, note non-saturation in recovery; (*e*) G2. Arrows indicate photobleaching event. (*f*) Summary of approximate *t*_1/2_ measurements at different cell-cycle phases and at DNA damage sites. (*g*) Analysis of recruitment of PCNA (mRFP-PCNA, red) and CENP-S (eGFP-CENP-S, green) following UV laser-induced focal DNA damage. (*h*) FRAP recovery of eGFP-CENP-S and mRFP-PCNA at sites of DNA damage, showing rapid recovery (*t*_1/2_ ∼ 160 ± 20 s) of CENP-S. (*i*) FRAP recovery of EGFP-CENP-S at centromeres, showing no recovery over a short-term time course.

The CENP-T/-W complex exhibits dynamic interaction with centromeres *in vivo*, in contrast to that observed for CENP-A [14,29]. To determine what type of binding mediates CENP-S and -X association with centromeres, fluorescence recovery after photobleaching (FRAP) was applied to FP-tagged centromeric CENP-S and -X during defined cell-cycle stages. Subunit exchange was examined as well as at sites of DNA damage. Cells expressing FP constructs at levels suitable for measurement were chosen for analysis to minimize perturbation to the protein pool. Short-term (6 min time course) FRAP was analysed in G1-, early S-, mid-S-, late S- and G2-phase cells identified by PCNA localization, revealing an absence of recovery of CENP-S in this time frame irrespective of which terminus was tagged (EGFP-CENP-S shown in figure 4*i*; electronic supplementary material, figure S4A). In order to detect slow CENP-S exchange at kinetochores, long-term FRAP (4 h) was carried out in cell-cycle phases defined at the prebleach time point using C-terminally tagged CENP-S-EGFP to ensure all EGFP signal derived from fully translated protein (figure 4*a*–*e*). At least four cells in each cell-cycle phase were analysed at 30 min time intervals for 4 h to determine the relative intensity of fluorescence following a bleach event (at *t* = 20 s). In each cell, the fluorescence intensity of five kinetochores located in bleached area was compared with five kinetochores in an unbleached area (electronic supplementary material, figure S5). In all cells examined, CENP-S-EGFP exhibited fluorescence recovery, to an extent of 90% or more. We assume that the CENP-S-EGFP fraction exchanges completely without an immobile protein fraction. In G1, the half recovery time, *t*_1/2_, is 90 ± 20 min, while in all other cell-cycle phases *t*_1/2_ is slower at 120 ± 20 min (figure 4*f*). The reproducibility of measurements indicates that consistent conditions are established in this transfection model but it is noted that the quantitative values may be affected by protein overexpression. Notably, recovery curves approximated an asymptotic approach to 100% in all cell-cycle phases except late S (figure 4*d*), where recovery showed no sign of reaching saturation. This is consistent with *de novo* assembly occurring in this phase of the cell cycle, as indicated by CLIP-labelling experiments described above.

In order to compare CENP-X dynamics with that of CENP-S, EGFP-CENP-X was analysed by FRAP in S phase (electronic supplementary material, figure S6). Surprisingly, we found a much higher mobility of EGFP-CENP-X at centromeres compared with EGFP-tagged CENP-S. The estimated half-time of fluorescence recovery of EGFP-CENP-X in early S phase (*t*_1/2_ ≈ 3 min) is shorter than in mid- and late S phase (*t*_1/2_ ≈ 9 min). Thus, at centromeres CENP-X binds to CENP-S by moderately fast exchange indicating that CENP-S is more stably bound at the kinetochore than CENP-X.

### 3.5. CENP-S interaction with sites of DNA damage occurs through a distinct binding mode

CENP-S and -X also play a role in DNA damage repair in association with the FANCM complex [35,36]. To determine whether binding to these sites is similar to the binding reactions at centromeres, CENP-S behaviour after DNA damage was analysed in live cell experiments. A HeLa cell line expressing mRFP-PCNA was transfected with EGFP-CENP-S. Two days later, UV_349nm_-mediated DNA damage was induced in a 0.2 µm^2^ area within the nucleus followed by recording of EGFP fluorescence. PCNA was rapidly recruited while EGFP-CENP-S was more slowly (*t*_1/2_ ≈ 20 min) recruited to the DNA damage site, reaching a plateau after approximately 40 min (figure 4*g*). CENP-S dwelled at DNA damage foci for a few hours and was no longer detectable after approximately 4 h. These results are similar to data obtained for endogenous CENP-S using immunofluorescence, indicating that after UV-mediated DNA damage, EGFP-CENP-S behaves as endogenous CENP-S [36].

EGFP-CENP-S FRAP experiments were then carried out to examine dynamics of protein binding at DNA damage sites. For EGFP-CENP-S localized at DNA damage foci, we observed a half-time of fluorescence recovery of *t*_1/2_ = 160 ± 20 s irrespective of the cell-cycle phase and time after UV-damage (figure 4*h*). This value is much faster than the half-time of EGFP-CENP-S recruitment to the DNA damage site (*t*_1/2_ ≈ 20 min), indicating that the accrual of CENP-S at DNA damage sites is not limited by rates of binding. This is also in stark contrast to the EGFP-CENP-S exchange at kinetochores (*t*_1/2_ ≈ 90–120 min). CENP-S total fluorescence recovery at DNA damage sites varied from 35 to 60%, indicating that about half of the EGFP-CENP-S bound at DNA damage sites is immobile. At these sites, by live cell imaging, we did not detect CENP-T. Taken together, these experiments clearly show that the mechanisms of CENP-S binding to centromeres and DNA damage sites are distinct, suggesting that the architecture of the binding site for the CENP-S/-X complex determines its mode of binding.

### 3.6. Centromeric binding site of the CENP-S/X complex

**Figure 5.**
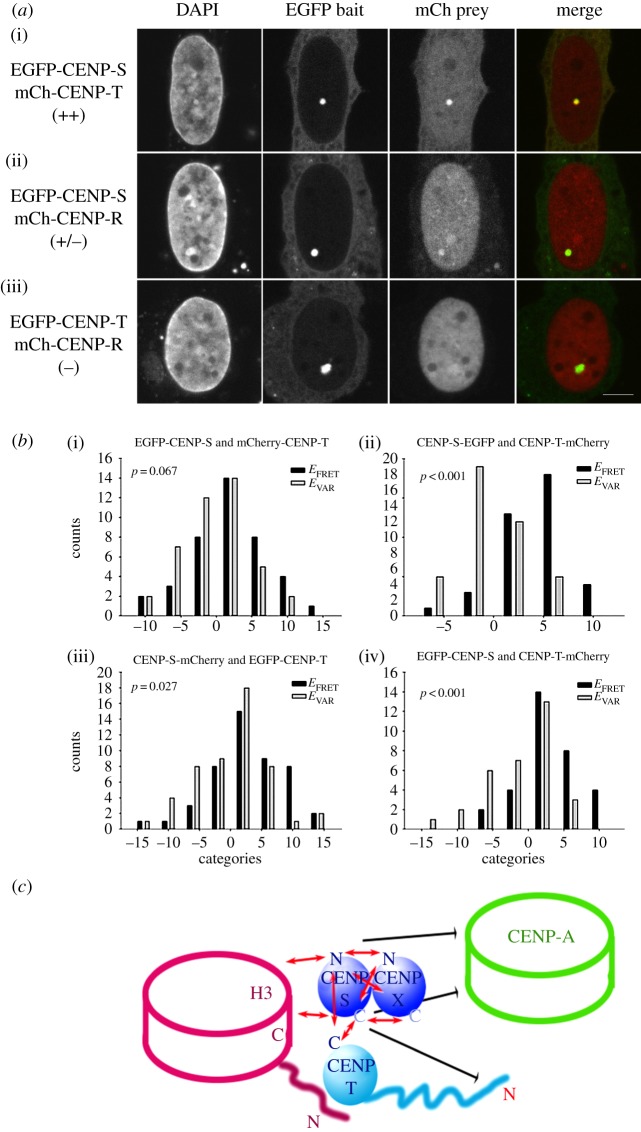
Centromere protein interactions analysed by F2H and FRET assays. (*a*) EGFP-mCherry fluorescent hybrid analysis of CENP-S interactions. EGFP-tagged centromere proteins were recruited to the *lac*-operator repeat array as bait by the GFP-binding protein fused to the Lac repressor (LacI-GBP) forming a green spot in the nucleus, stained by DAPI. Co-expressed mCherry-tagged centromere proteins (prey) may either interact with the GFP-tagged protein at the *lac-*operator array (visible as red spot and yellow in the overlay) or may not interact resulting in a disperse distribution. A strong interaction was observed between EGFP-CENP-S and mCherry-CENP-T (i). While mCherry-CENP-R interacts with EGFP-CENP-S weakly (ii), there is no interaction between mCherry-CENP-R and EGFP-CENP-T (iii). For each mCherry fusion, EGFP was used to control for unspecific interactions. The bar represents 5 μm. (*b*) FRET reveals CENP-T/W/S/X particle assembly proximal to histone H3. Bar diagram of *in situ* acceptor photobleaching-based FRET measurements between fluorophore-tagged CENP-S and CENP-T. FRET efficiency values within the bleached region were classified into 4% deviating categories (*x*-axis) resulting in *E*_FRET_ (black bars). As control, FRET efficiency values in the same nucleus at centromeres, where mCherry was not bleached, were also categorized resulting in *E*_VAR_ (grey bars). On *y*-axis, the number of counts for each category is displayed. Mann–Whitney rank-sum test was used to determine *p*-values as indicator for differences between the input groups (i.e. all *E*_FRET_ and *E*_VAR_ values). Examples are shown for CENP-S:CENP-T interaction analysis. No interaction between CENP-S and an N-terminally labelled CENP-T probe was observed (FP-CENP-S (i) and CENP-S-FP (iii)). Positive FRET was observed between either CENP-S probe and a CENP-T-FP probe ((ii) and (iv)). (*c*) Diagrammatic representation of FRET analysis. Red arrows indicate positive FRET interaction signal, while black arrows signify a lack of signal detected. The CENP-T/W/S/X particle shows intraparticle FRET throughout the network, as expected for proteins of this size. CENP-S interacts with C-terminally labelled histone H3, but not with CENP-A.

Examination of the assembly and binding of the CENP-S/-X complex reveals that its centromere association is specifically regulated during the cell cycle and that its mode of binding to centromeres is distinct from that observed at sites of DNA damage. To ask which centromere components could be associated with this binding, we applied two-hybrid assays to characterize the interactions of CENP-S with CENPs -A, -M, -R and -T. Using yeast two-hybrid assays, we could not detect any interaction of CENP-S with other CENPs. However, using a mammalian fluorescent two-hybrid assay (F2H) based on a *lac* repressor array [46], specific interactions were observed (figure 5*a*; electronic supplementary material, table S3). In this assay, a GFP-CENP fusion protein bait is tethered to an integrated *lac*-operator array and its ability to recruit other mRFP-CENP fusion proteins is analysed, though it is not clear that recruitment is through direct interactions. CENP-S showed strong and slightly less strong interactions with CENP-T and CENP-R, respectively, while CENPs -A and -M indicated weaker or no co-localization.

To examine the interactions of the CENP-S/-X complex at centromeres *in vivo*, acceptor-bleaching (AB)FRET methods were applied with other proteins known to be resident in centromeric chromatin. EGFP-fluorescence at centromeres was measured before and after bleaching an mCherry test protein in a 4–10 µm^2^ region of the nucleus. At least 30 centromeres were analysed for each complete FRET measurement. Although CENP-S and CENP-R showed weak F2H interaction, we could not detect FRET between the tagged variants at the human kinetochore. Positive FRET was observed between CENP-S labelled at either terminus and the C-terminal domain of CENP-T (figure 5*b*). By contrast, the CENP-T N-terminus showed no or non-significant FRET with both CENP-S termini (see table 1). These FRET data support the F2H results discussed above and confirm that the CENP-S/-X interaction with CENP-T shown by biochemical and crystallographic methods [30,34] can be detected *in situ* at centromeres.

CENP-T/-W has been reported to interact specifically with histone H3 nucleosomes within centromeric chromatin rather than with CENP-A [9]. To determine which nucleosomal compartment the CENP-S/-X complex is most closely associated with *in vivo*, the proximity of CENP-S to histone H3 and to CENP-A was analysed by FRET. These experiments revealed close proximity of CENP-S to the C-terminus of histone H3.1, but not to CENP-A (table 1; diagrammed in figure 5*c*). Thus, these results are consistent with structural studies and confirm a series of immunoprecipitation experiments that show interactions among the CENP-T/W/S/X network, documenting the preferential interaction of the novel nucleoprotein particle with histone H3 nucleosomes *in vivo*.

## 4. Discussion

The CENP-S/-X complex is part of a unique histone fold particle that participates in kinetochore assembly and in DNA repair processes [15,30,34–36,47,48]. Here, we studied CENP-S/-X assembly in living human cells, revealing a window in S phase and G2 in which *de novo* assembly of the complex from a soluble precursor occurs through a dynamic exchange mechanism. CENP-S is not found in a soluble complex with its binding partner CENP-T but it interacts strongly and specifically with immobilized CENP-T in an *in vivo* binding assay. The *in vivo* FRET analysis reported here reveals co-assembly of CENP-S and -X with CENP-T at centromeres in proximity to histone H3, but not to CENP-A, consistent with the structure and biochemistry of this complex reported by others [30,34]. CENP-T and -W assemble in the same time frame during the cell cycle [14]. These assembly events point towards a distinct kinetochore-associated chromatin assembly pathway that operates after DNA replication at centromeres, perhaps coordinated with histone H3 nucleosome assembly events [26].

### 4.1. Centromeric chromatin

**Figure 6.**
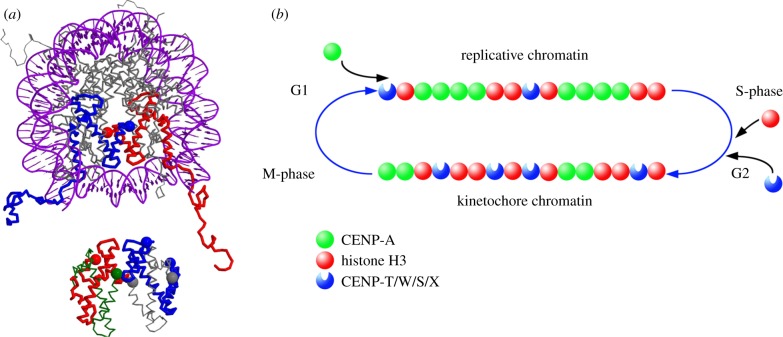
Model of CENP-T/W/S/X in chromatin. (*a*) Potential position of the CENP-TWSX tetramer relative to a classical nucleosome. Top: top view of the nucleosomal structure including DNA (1kx5, [49]) with both H3 molecules coloured in red and blue, respectively; the H3 C-termini are indicated by coloured spheres. Bottom: structure of the CENP-TWSX tetramer (3vh5; [30]) positioned in the plane relative to the nucleosomal structure. The tetramer is assumed to be partly surrounded by DNA, and accordingly, space is left between the two structures (2 nm). Colour coding: CENP-T, red with C-terminus indicated by a large sphere; CENP-W, green with C-terminus indicated by a large sphere; CENP-S, blue with both termini marked with a large sphere; CENP-X, grey with both termini marked with a large sphere. Only well-determined termini positions are shown. The largest distance of 8.4 nm is found between the H3 C-terminus and the CENP-X N-terminus, thus all termini shown are sufficiently proximal for FRET. Note the close proximities of the CENP-S and -X termini, explaining well the measured FRET. Shown is one potential positioning of the two complexes, also alternative arrangements (for example, the CENP-TWSX tetramer between two classical nucleosomes) would explain well the measured FRET results. (*b*) Proposed switching mechanism for converting centromeric chromatin between functional states. Completion of CENP-A assembly results in replication-competent chromatin, top, which is segregated to daughter chromatids during S phase. Assembly of CENP-T/W/S/X, probably in conjunction with histone H3, is activated by DNA replication, resulting in kinetochore-competent chromatin, bottom, with a distinctive subunit composition for function in mitosis.

Our FRET proximity analysis of CENP-T/W/S/X proteins and the H3 C-terminal domain constrains the relative positioning of these two complexes: the CENP-T/W/S/X complex might lie close to the DNA exit of the H3 nucleosome (see figure 6*a*) or partly in between two H3 nucleosomes (not shown). We consider the path of the DNA in the centromeric complex still unresolved: either all three nucleosomal particles (the two either CENP-A or H3-containing nucleosomes and the CENP-T/W/S/X tetramer) lie in *cis* along the chromosomal DNA strand, or alternatively, proximity is established by the folding of the chromatin fibre that brings regions distant with respect to DNA into contact in *trans*.

Assembly of CENP-S and -X and its partner subunits CENP-T and -W occurs through a dynamic exchange mechanism. The distinct kinetics of CENP-S/-X interaction with centromeres versus sites of DNA damage is consistent with the different structures proposed for CENP-S/-X hetero-tetramers versus centromeric CENP-T/W/S/X particles [30], and separation of centromeric and DNA damage functions has been shown for *Schizosaccharomyces pombe* CENP-S and -X homologues [48]. At centromeres, CENP-T and -W exhibit FRAP recovery half-times of approximately 60 min [14] while CENP-S exchanges with approximately 120 min half-time. CENP-X exhibits very dynamic association with the centromeric complex, with a turnover half-time of approximately 10 min during S phase. Thus, the complex has a distinct kinetic signature compared with normal or CENP-A nucleosome components, in which core tetramer half-lives are measured in the framework of hours [50] or generations in the case of CENP-A [24,25]. The results indicate that although exchange is continuous in the cell cycle, *de novo* assembly is only observed in mid–late S and G2 phase cells. The observed assembly in S and G2 phases probably represents a combination of increase in the number of binding sites as well as the affinity of the complex. Alternatively, or as well, there could be a difference in the activity of G1 versus S/G2 pools of soluble protein and a distinct loading reaction with similar kinetics is responsible for *de novo* assembly. Attempts to directly measure affinity with photoswitching proteins were unsuccessful. Taken together, these results suggest that the uniquely dynamic CENP-T/W/S/X chromatin subunit may be particularly adapted to the open, self-organizing environment of the mitotic spindle.

### 4.2. Just-in-time mode of assembly

Examination of transcript profiles for CCAN components has, in general, revealed little evidence for strong transcript-level control in this family of proteins (N Quinn and KF Sullivan 2011, unpublished data). Rather, we propose, assembly is initiated by key events, such as DNA replication and mitosis, and is probably driven by post-translational controls [51], nucleocytoplasmic transport [52] as well as control of protein abundance in S phase and G2. The open exchange observed for most CCAN components [14,29,53,54] indicates that the composition of the kinetochore is a steady-state configuration rather than a fixed architecture, such as a centriole or nuclear pore. Tethering experiments in which CCAN components CENP-T and -C [12,15], HJURP [7] or CENP-A itself [6] are used as initiators invariably result in the formation of microtubule-binding kinetochore-like structures in mitosis, demonstrating that the endpoint can be reached from a variety of starting configurations. The open, adaptive potential of stochastic, just-in-time type ‘triggered’ assembly mechanisms has been suggested to stabilize cell-cycle-dependent pathways by providing ways for translational and post-translational mechanisms to evolve while maintaining the functional outcome of timely assembly [55]. A just-in-time mode of kinetochore assembly provides a dynamic, open chromosomal interface for the highly stochastic mechanisms of microtubule binding, dynamic motility and error correction that are necessary for a robust mitosis [1].

### 4.3. Switching model

These results suggest that the centromere naturally cycles between two functional states, typified by distinct nucleosome/nucleoprotein composition and ‘primary structural’ organization (figure 6*b*). A multistep pathway for CENP-A deposition has been described in which CCAN components interact both with CENP-A and with CENP-A-HJURP complexes to initiate CENP-A assembly in telophase [7,18,19,56,57]. CENP-A assembly occurs in G1 and involves RSF-dependent remodelling and a G protein-dependent stabilization reaction [27,28]. This extended CENP-A chromatin assembly process leads to a fully assembled CENP-A chromatin structure at centromeres at the end of G1. A critical role for CENP-T in kinetochore formation through its interaction with Ndc80 has been documented by tethering and structural studies. The assembly of the CENP-T/W/S/X particle documented here and by Prendergast *et al.* [14] suggests the existence of a functional chromatin assembly pathway that operates in complement to the CENP-A pathway to prepare the centromere for kinetochore formation and function. In this model, the product of the CENP-A cycle is a ‘replication-competent’ centromeric chromatin. During the next major cell-cycle event, DNA replication, these CENP-A nucleosomes will be passed to daughter chromatids with 100% efficiency [24,25]. S phase initiates a chromatin pathway that specifically enriches CENP-T/W/S/X at centromeres, probably in association with histone H3.1 and 3.3, both shown to assemble at centromeres at this time in *Drosophila* [26]. The resulting ‘kinetochore-competent’ chromatin is specifically adapted to mitosis through this reprogramming of its component particles. In addition to the nucleosome-like CENP-T/W/S/X particle, much of the CCAN assembles during this time period, including the CENP-P/O/R/Q/U subcomplex and CENP-N [53,54]. Unlike CENP-A, whose presence is strictly conserved at centromeres [25], the CENP-T/W/S/X particle and most CCAN-binding components are in dynamic equilibrium with the centromere, some in direct coupling to kinetochore functional state [58]. We propose that segregation of CENP-A and kinetochore chromatin-associated assembly events promotes integration of the stability required of an epigenetic transmitter with the dynamic, stochastic nature of the kinetochore in mitosis.

## 5. Material and methods

### 5.1. Cell culture

HeLa and U2OS cells were cultured in DMEM with 10% FCS, as previously described [51,59]. Cell synchrony using the double-thymidine protocol was as described by Prendergast *et al.* [14].

### 5.2. Plasmids

Plasmids pIC235, pDF180, pDF197, encoding LAP-CENP-R, -S and -T fusions proteins, respectively, were a kind gift of Dan Foltz and Iain Cheeseman. CENP-X was obtained directly by PCR from cDNA prepared from HeLa total RNA. Full-length coding sequences were amplified by PCR (Expand high fidelity^PLUS^ PCR System, Roche, Penzberg, Germany) using primers incorporating flanking attB recombination sites and transferred into vector pDONR221 by BP recombination reaction (Invitrogen, Carlsbad, CA, USA; electronic supplementary material, table S1). Genes were then transferred by LR recombination reactions into modified pFP-C- and pFP-N (BD Biosciences, Clontech, Palo Alto, CA, USA)-based Destination vectors. CLIP-tagged proteins were generated by recombination into a Gateway modified pCLIPm vector (New England Biolabs, Isis Ltd, Bray, Co. Wicklow, Ireland) as described by Prendergast *et al.* [14]. All constructs were verified by DNA sequencing (MWG Biotech, Ebersberg, München, Germany).

### 5.3. Cell lines and transfection

Stably transfected cell lines expressing CENP-S-CLIP and CENP-X-CLIP were established in HeLa cells by electroporation using a NucleofectorII device (Lonza Biologics, Slough, UK). One CENP-S-CLIP and three independent CENP-X-CLIP cell lines were used for all experiments in this study. For FRAP, FRET and FCCS experiments, appropriate constructs were transfected into HeLa and U2OS cells by electroporation and assayed after 24–48 h. FRAP experiments were performed using a HeLa cell line that stably expresses mRFP-PCNA [60].

### 5.4. Western blot and real-time PCR

For protein detection, whole-cell extracts equivalent to 50 000 cells were separated by SDS-PAGE, transferred to PVDF membranes (Millipore) and processed for immunodetection, as previously described [14]. Antibodies are specified in the electronic supplementary material, table S2. Following detection by ECL (Millipore Ireland B.V., Tullagreen Carrigtwohill, County Cork, Ireland), blots were imaged using a Syngene G:BOX imager (Mason Technologies, Dublin, Ireland) and quantitated using Image J (http://rsb.info.nih.gov/ij/). Relative protein abundance was calculated as the mean intensity normalized to tubulin and is presented as the mean ± s.e.m. (*n* = 3 independent experiments).

Total RNA was isolated using Qiagen RNeasy Mini Kits (Qiagen, Crawley, West Sussex, UK) and reverse transcribed using random nonamer primers with a Precision qScript Reverse-Transcript Kit supplied by Primer Design (Southampton, UK). Quantitation of transcripts was performed using SYBR green reagents (Primer Design) on a StepOnePlus Real-Time PCR System (Life Technologies, Paisley, UK). Primers were prepared by Primer Design and are specified in the electronic supplementary material, table S1. Primer efficiencies were determined empirically and specificity was verified by DNA sequence analysis of PCR products. The endogenous control used in this study (GAPDH) was determined to be optimal using the GeNorm kit and software (Primer Design). Target abundance was calculated from qPCR data using the Pfaffel method, which incorporates specific primer efficiencies in calculation of ΔΔCT values, and the relative fold changes (relative quantitation, RQ) were then calculated by the formula, *E*^−ΔΔCT^ where *E* is the primer efficiency [61].

### 5.5. Immunofluorescence and CLIP labelling

Immunofluorescence and CLIP labelling were performed essentially as described previously [14] using antibodies specified in the electronic supplementary material, table S2 and reagents supplied by New England Biolabs (ISIS Ltd, Bray, Co. Wicklow, Ireland). For direct quantitation of CLIP-tagged proteins, cells grown on coverslips were labelled with 2 mM CLIP-505 in complete DMEM supplemented with 1% BSA for 45 min prior to washing and fixation. For quench-pulse labelling, CLIP-tagged proteins were first blocked with CLIP cell block at 10 mM in DMEM supplemented with 1% BSA for 30 min. After washing, cells were returned to the incubator and cultured for 8 h prior to labelling with CLIP-505.

### 5.6. F2H cell culture and transfections

BHK cells containing a *lac*-operator repeat array [62] were cultured in DMEM medium with 10% FCS and seeded on coverslips in six-well plates for microscopy. After attachment, cells were co-transfected with expression vectors for the indicated fluorescent fusion proteins and a LacI-GBP fusion [46] using polyethylenimine (Sigma, St. Louis, MO, USA). After about 16 h cells were fixed with 3.7% formaldehyde in PBS for 10 min, washed with PBST (PBS with 0.02% Tween), stained with DAPI and mounted in Vectashield medium (Vector Laboratories, Servison, Switzerland). Samples were analysed with a confocal fluorescence microscope (TCS SP5, Leica, Wetzlar, Germany) equipped with a 63×/1.4 numerical aperture Plan-Apochromat oil immersion objective, as described [46].

### 5.7. Fluorescence cross-correlation spectroscopy

FCCS analyses [63] were performed at 37°C on an LSM 710 Confocor3 microscope (Carl Zeiss, Jena, Germany) using a C-Apochromat infinity-corrected 1.2 NA 40× water objective. U2OS cells were double transfected with vectors for the simultaneous expression of EGFP- and mCherry-fusion proteins and analysed. On cells expressing both fusion proteins at relatively low and comparable levels, we selected spots for the FCCS measurements in areas of the nucleoplasm that were free of kinetochores. EGFP-fusion proteins were excited using the 488 nm laser line of a 25 mW Argon/2-laser (Carl Zeiss) and mCherry-fusion proteins with a DPSS 561-10-laser (Carl Zeiss), both at moderate intensities between 0.2 and 0.5%. The detection pinhole was set to a relatively small diameter of 40 µm. After passing a dichroic beam splitter for APDs (avalange photodiode detectors; NTF 565), the emission of mCherry was recorded in channel 1 through a BP-IR 615–680 nm bandpath filter by an APD (Carl Zeiss), whereas the emission of EGFP was simultaneously recorded in channel 2 through a BP-IR 505–540 nm bandpath filter by the second APD. Before each measurement, we analysed possible crosstalk between the channels and used only cells without or with very little crosstalk. In addition, measurements with autocorrelation values below 1.06 for both the mRFP and EGFP channels were not further analysed. For the measurements, 10 time series of 10 s each were simultaneously recorded for mCherry and EGFP. After averaging, the data were superimposed for fitting with the Fit-3Dfree-1C-1Tnw model of the ZEN-software (Carl Zeiss), a diffusion model in three dimensions with triplet functions.

### 5.8. Acceptor photobleaching-based FRET measurements

FRET experiments were conducted, as described previously by Orthaus *et al*. [40] and Hellwig *et al*. [53]. EGFP-fluorescence before and after mCherry-bleaching of a 4–10 µm^2^ region of interest that contained 2–5 centromeres was compared, resulting in FRET efficiency (*E*_FRET_) values. Additionally, EGFP fluorescence in an unbleached area that contained an equal amount of centromeres (±1) was analysed resulting in control FRET efficiency (*E*_VAR_). The determined *E*_FRET_ and *E*_VAR_ values were classified into 4% deviating categories and the number of counts for each category is shown in a bar diagram. Both input groups (*E*_FRET_ and *E*_VAR_ values) were statistically evaluated using Mann–Whitney rank-sum test.

### 5.9. Fluorescence recovery after photobleaching

FRAP experiments were carried out on a Zeiss LSM 510Meta confocal microscope (Carl Zeiss) using a C-Apochromat infinity-corrected 1.2 NA 63× water objective and the 488 nm laser line for GFP, essentially as described previously [14,51]. Measurements were made by transfection of the indicated FP constructs into HeLa cells stably transfected with mCherry-PCNA. Five or 10 images were taken before the bleach pulse and 50–200 images after bleaching of two to four kinetochores of a nucleus with an image acquisition frequency of 0.5–1.0 frames per second at 1% laser transmission to avoid additional bleaching. In long-term FRAP experiments, the pinhole was adjusted to 1 airy unit and image stacks were taken every 30 min. Relative fluorescence intensities were quantified as described by Chen & Huang [64] and Schmiedeberg *et al*. [65] using Excel (Microsoft, Redmond, WA, USA) and Origin software (OriginLab, Northampton, MA, USA).

## Supplementary Material

Electronic Supplementary Materials REVISED-Dornblut_Quinn
