# Supplementary material for: A CENP-S/X complex assembles at the centromere in S and G2 phases of the human cell cycle
Source: Open Biol. 2014 Feb 12;4(2):130229. doi: 10.1098/rsob.130229 (PMC3938055; doi:10.1098/rsob.130229)
Supplement: Electronic Supplementary Materials REVISED-Dornblut_Quinn [file rsob130229supp1.pdf]

Supplementary Figure S1

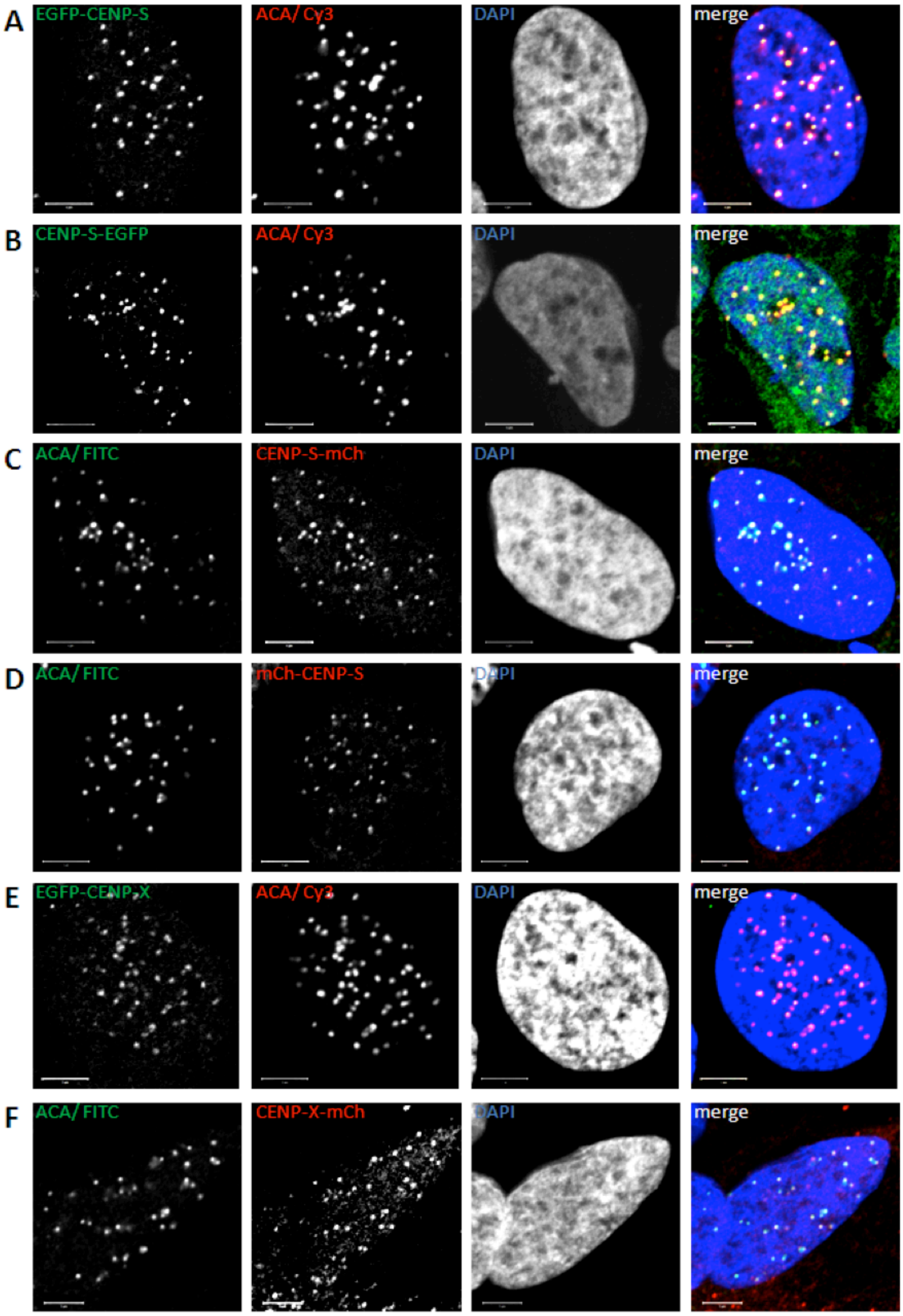

Figure S1. **CENP-FP constructs target to centromeres irrespective of site of tagging.** FP-CENP and CENP-FP constructs were transfected into U2OS cells and counterstained with anti-centromere antibody (ACA) in the appropriate color channel. A) EGFP-CENP-S; B) CENP-S-EGFP; C) CENP-S-mCh; D) mCh-CENP-S; E) EGFP-CENP-X; F) CENP-X-mCh. Scale bar equals 5 microns.

Supplementary Figure S2

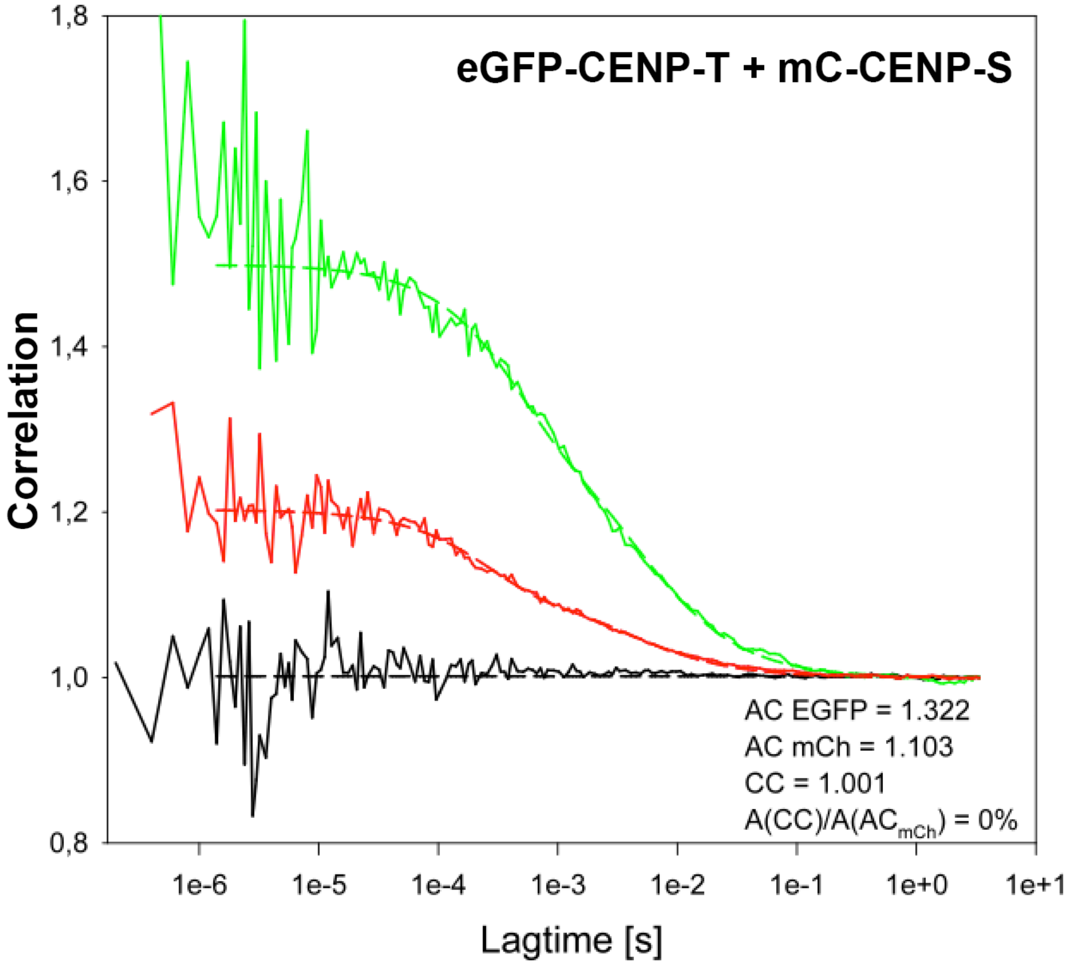

Figure S2**. FCCS analysis of CENP-T and CENP-S.** EGFP-CENP-T and mCh-CENP-S were co-expressed and analyzed as in Figure 1. Correlation spectra for EGFP-CENP-T (green) and mCh-CENP-S (red) show no cross-correlation (black).

Supplementary Figure S3

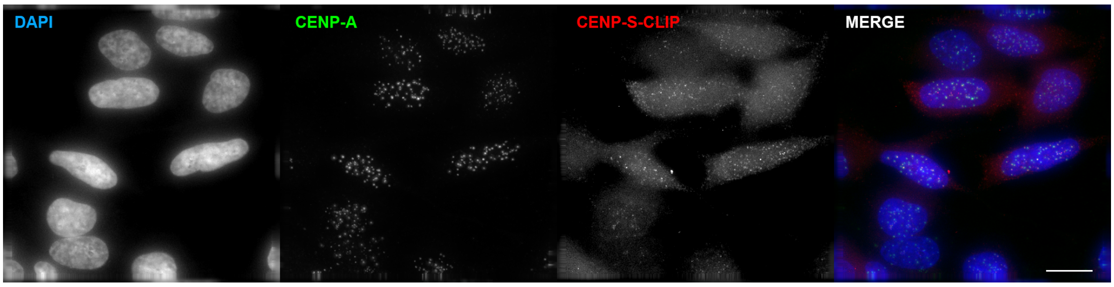

Figure S3. **CLIP-505 labeling of CENP-S-CLIP**. Asynchronous cells were fixed and immunostained for CENP-A (green in merge). Cells were also labelled with CLIP-505 to reveal CENP-S localization (red in merge). DNA was stained with DAPI (blue). Scale bar equals 10 microns.

Supplementary Figure S4

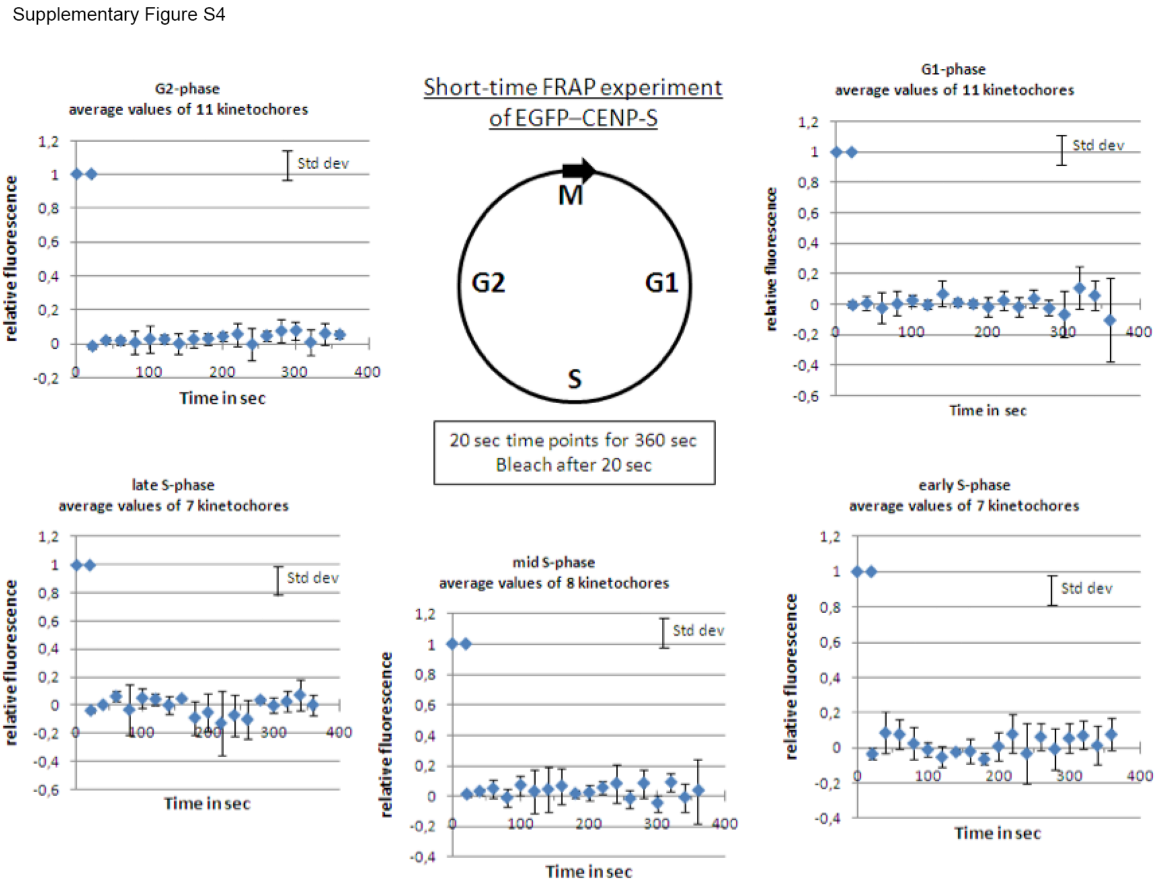

Figure S4. **Short term FRAP with EGFP-CENP-S.** Short-term FRAP with EGFP-CENP-S at different cell cycle stages. HeLa cells expressing mRFP-PCNA were transiently transfected with EGFP-CENP-S and cells were staged on the basis of PCNA pattern and nuclear size. At each stage, the indicated number of kinetochores were examined in at least 3 cells. Data are reported as the mean with standard deviations. No significant recovery in a 360 second time course was observed.

Supplementary Figure S5

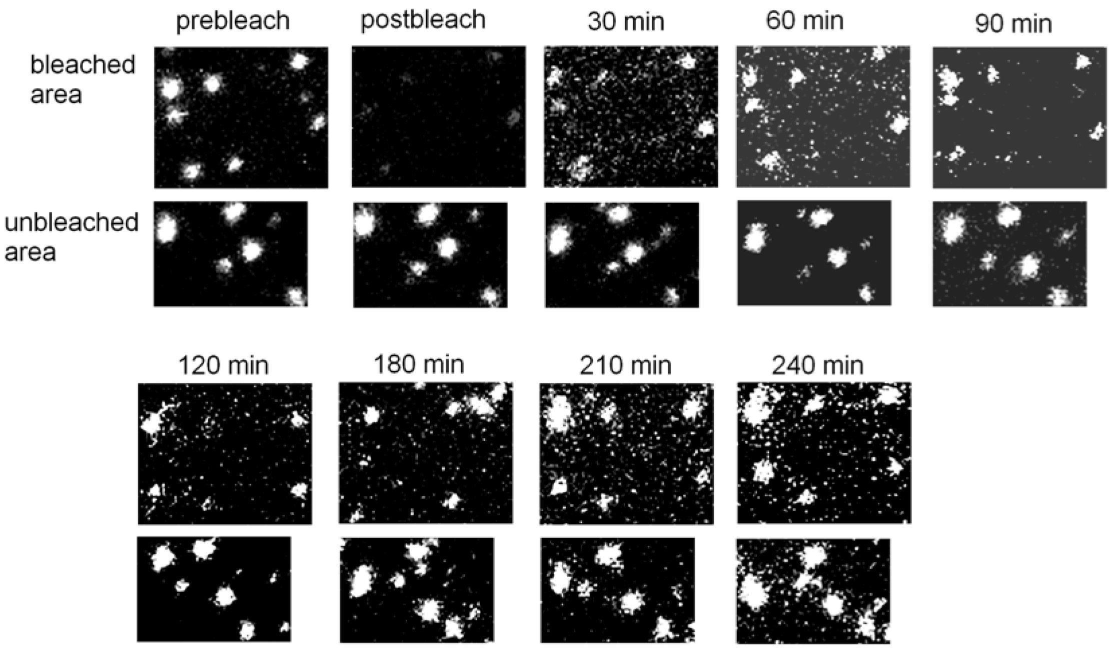

Figure S5. **Example of long-term CENP-S-EGFP FRAP recovery at centromeres**. FRAP analysis in a G1 cell is presented. Two small fields are shown at each of a series of time points, containing equivalent kinetochore numbers. The upper field was bleached at the indicated time and images were collected at consecutive 30 minute intervals. The lower field is a control, unbleached region. Recovery approaches 100% in the bleached zone.

Supplementary Figure S6

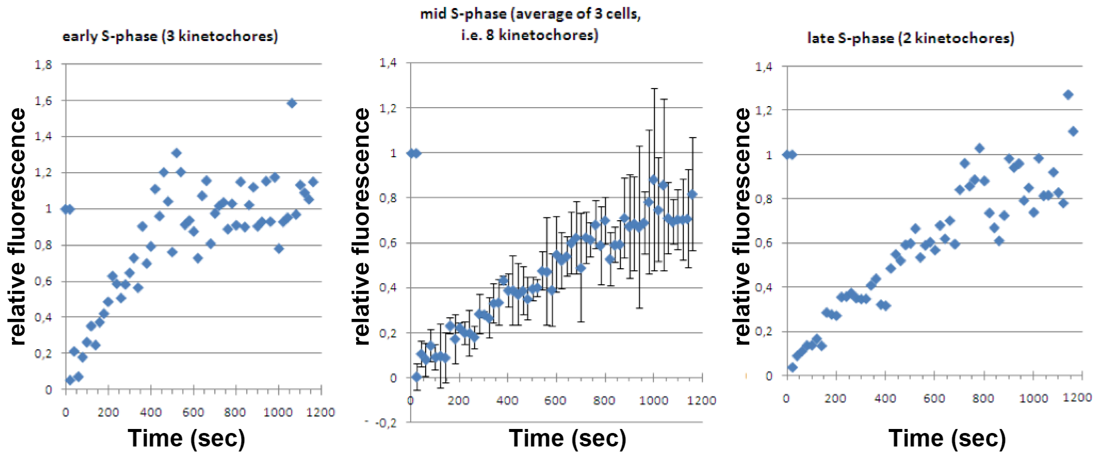

Figure S6. **FRAP with CENP-X.** HeLa cells expressing mRFP-PCNA were transiently transfected with EGFP-CENP-X and cells were staged in S-phase on the basis of PCNA pattern and nuclear size. FRAP was performed as described on the indicated number of kinetochores. CENP-X recovery was rapid and approached 100%.

Supplementary Table 1: Primers used

Primer Set	Sense Primer,	Antisense Primer,	Product
CENP-A_{RT}	CGCTTCCTCCCATCAACAC	GGGCAGCTTCCTTATCAAGAG	qPCR product
CENP-B_{RT}	GAAGCCCAGTGCCCTACTC	TCCTCATCATCATCGTCGTCTT	qPCR product
CENP-C_{RT}	AGTGAATCCAGTCCCATTGTTAG	TCATCCTCTATCAACTTCGTATCAT	qPCR product
CENP-S_{RT}	CGCAGTGATGGAGGAGGAG	CGCAAAGACAACCCACAGTAT	qPCR product
CENP-X_{RT}	CCTCCTGGCCACATTCCTG	GATTTATTGATGTTGCTTTGTGAGAA	qPCR product
Cyclin A_{RT}	ACAGCCAGACATCACTAACAG	CAGCACTGACATGGAAGACA	qPCR product
Cyclin B_{RT}	AGCAAGCAGTCAGACCAAAAT	CAGTCAATTAGGATGGCTCTCA	qPCR product
CENP-R	GGGGACAAGTTTGTACAAAAAAGCAGGCTTCGA AAACCTGTATTTTCAGGGCGCCACCATGGGCAT GCCTGTTAAAAGATCACTGAA	GGGGACCACTTTGTACAAGAAAGCTGGGTG TTTAAAATGGCTTTAAGGAATTCA	Full length CDS
CENP-S	GGGGACAAGTTTGTACAAAAAAGCAGGCTTCGA AAACCTGTATTTTCAGGGCGCCACCATGGAGGA GGAGGCGGAGAC	GGGGACCACTTTGTACAAGAAAGCTGGGT ATTCTCACTTTCCACCACTCCAGC	Full length CDS
CENP-T	GGGGACAAGTTTGTACAAAAAAGCAGGCTTCGA AAACCTGTATTTTCAGGGCGCCACCATGGCTGA CCACAACCCTGAC	GGGGACCACTTTGTACAAGAAAGCTGGGTC TGGGCAGGGAAGACAGAGTT	Full length CDS
CENP-X	GGGGACAAGTTTGTACAAAAAAGCAGGCTTCGA AAACCTGTATTTTCAGGGCGCCACCATGGAGGG AGCAGGAGCTGGAT	GGGGACCACTTTGTACAAGAAAGCTGGGTG AAGTCCAGGAGCAGCTGCG	Full length CDS
CENP-S D92-138	GGGGACAAGTTTGTACAAAAAAGCAGGCTTCGA AAACCTGTATTTTCAGGGCGCCACCATGGAGGA GGAGGCGGAGAC	GGGGACCACTTTGTACAAGAAAGCTGGGTT GAATTACTCCTCCTGGCTAAGAGC	N terminal HFD of CENP-S

Supplementary Table 2: Antibodies used.

Reactivity	Species	Conjugation	IF	WB	Supplier
CENP-A	Mouse IgG		1/300	1/1000	Abcam
CENP-B	Mouse IgG		-	1/250	W.Earnshaw
CENP-S	Rabbit IgG		-	1/500	W.Wang
CENP-X	Rabbit IgG		-	1/500	W.Wang
CENP-T	Rabbit IgG		-	1/1000	Bethyl Labs
CENP-W	Rabbit IgG		-	1/1000	Abcam
Cyclin B	Mouse IgG		-	1/2000	Upstate
Histone H3S10P	Rabbit IgG		-	1/2000	Millipore
PCNA	Human IgG		1/100	-	KF.Sullivan
Zwint	Rabbit IgG		1/100	-	Bethyl
Secondary antibodies:					
Anti-Rabbit IgG	Donkey	Cy5	1/200	-	Jackson Labs
Anti-Mouse IgG	Goat	TRITC	1/100	-	Jackson Labs
Anti-Human IgG	Donkey	AMCA	1/100	-	Jackson Labs
Anti-Rabbit IgG	Goat	HRP	-	1/10,000	Jackson Labs
Anti-Mouse IgG	Goat	HRP	-	1/10,000	Jackson Labs

Supplementary Table 3: Summary of F2H results

RFP \ GFP	CENP-A	CENP-M	CENP-R	CENP-S	CENP-T
CENP-A	-	-	-	-	-
CENP-M	-	-	+-	-	-
CENP-R	-	+-	-	+	-
CENP-S	-	-	+	-	++
CENP-T	-	-	-	++	-

++ denotes strong interaction
+ denotes detectable interaction
+- denotes weak interaction
- denotes undetectable interaction
